# Selective reduction of neurotransmitter release by cAMP-dependent pathways in mouse detrusor

**DOI:** 10.1152/ajpregu.00166.2022

**Published:** 2022-10-17

**Authors:** Basu Chakrabarty, Marcus J. Drake, Anthony J. Kanai, Christopher H. Fry

**Affiliations:** ^1^School of Physiology, Pharmacology, and Neuroscience, University of Bristol, Bristol, United Kingdom; ^2^Translational Health Sciences, Bristol Medical School, University of Bristol, Bristol, United Kingdom; ^3^Bristol Urological Institute, Southmead Hospital, Bristol, United Kingdom; ^4^Departments of Medicine and Pharmacology and Chemical Biology, University of Pittsburgh, Pittsburgh, Pennsylvania

**Keywords:** adenosine, adenosine triphosphate, cyclic adenosine monophosphate, detrusor smooth muscle, neurotransmitter release

## Abstract

Parasympathetic nerve-mediated contractions of detrusor smooth muscle are generated by ATP and acetylcholine (ACh) release from efferent nerve terminals. In humans, ACh is responsible for detrusor contractions in normal human bladders, whereas ATP has an additional role in overactive bladder pathologies. The ATP metabolite, adenosine, relaxes nerve-mediated contractions, with a potential action via presynaptic adenosine A_1_ receptor activation and subsequent suppression of neuronal ATP release. We investigated the effect of A_1_ receptor activation and downstream cAMP-dependent pathways on nerve-mediated ATP and ACh release, and detrusor contraction in mouse detrusor. Bladders from male C57BL/6 mice (12 wk) were used for in vitro experiments. Upon electrical field stimulation of intact preparations (detrusor and mucosal layers), ATP or ACh release was measured simultaneously with tension recordings. Activation of A_1_ receptors by adenosine or exogenous agonists reduced the lower frequency component of nerve-mediated contractions and neuronal ATP release. The A_1_ receptor antagonist abolished these effects. A_1_ receptor activation inhibits adenylyl cyclase (AC) activity and cAMP generation. The effect of A_1_ receptor activation was mimicked by a PKA antagonist but not by modulators of exchange proteins activated by cAMP, demonstrating that modulation of nerve-mediated ATP release is via PKA. Adenosine had no effect on ACh release or the higher frequency component of nerve-mediated contractions. Differential regulation of neurotransmitter release is possible at the detrusor nerve-muscle junction, as demonstrated by A_1_ receptor activation, and downstream inhibition of AC, cAMP generation, and PKA. The ability to specifically attenuate ATP release offers a potential to target purinergic motor pathways enhanced in overactive bladder pathologies.

## INTRODUCTION

The purinergic system regulates bladder function through the action of ATP and its metabolites. ATP release from bladder-efferent nerves plays a role in autonomic neurotransmission (1–3). It has been identified in sympathetic and parasympathetic varicosities, where ATP is coreleased with either noradrenaline or acetylcholine (ACh) (4). The contribution of ATP to parasympathetic nerve-mediated contractions in detrusor smooth muscle has been extensively studied and has a role in most animal bladders. However, in humans, ATP is a functional neurotransmitter only in pathological situations, such as overactive bladder (OAB) syndrome, detrusor overactivity (DO), or bladder outlet obstruction. Detrusor contractions from normal human bladders are solely supported by ACh (5–12). In consequence, the ability to selectively attenuate nerve-mediated ATP release offers itself as an attractive therapeutic target to manage clinical conditions.

Extracellular ATP is rapidly metabolized, ultimately to adenosine, by extracellular endonucleotidases (13). Adenosine itself may bind to surface receptors (A_1_, A_2A_, A_2B_, and A_3_), but has other fates such as translocation to the cytoplasm, or conversion to inosine by adenosine deaminase, or AMP by adenosine kinase (14–16): all of which have important roles in the urinary tract (17, 18). Actions of adenosine receptor activation are mediated by G protein-coupled intracellular pathways to modulate adenylyl cyclase (AC) activity and hence cAMP generation; A_1_ and A_3_ receptors inhibit AC activity, whereas A_2A_ and A_2B_ receptors stimulate AC activity. Downstream cAMP pathways include intermediates, such as PKA and exchange proteins activated by cAMP (EPACs) (19). One action of adenosine is to regulate neurotransmission at synapses or neuroeffector junctions where ATP participates as a cotransmitter, in addition to the fact that adenosine may itself be a neuromodulator (20, 21).

Overall, adenosine attenuates nerve-mediated contraction of detrusor from mice (20), rats (22, 23), guinea pigs (1, 24), and humans (6, 25). With human detrusor, this action is greater in tissue from patients with neurogenic DO (NDO) compared with normal stable bladders and is mimicked by the selective A_1_ receptor agonist *N*^6^-cyclopentyladenosine (CPA) (6). Moreover, with tissue from NDO bladders, there is a greater reduction of force by A_1_ receptor agonists at lower frequencies of stimulation (1–4 Hz), with a greater dependence on ATP release, compared with higher frequency contractions where ACh release is dominant (6). This has been interpreted as A_1_ receptor activation having a relatively selective action on nerve-mediated ATP release. However, immunolocalization confocal microscopy has demonstrated A_1_ receptors to be colocalized with vesicular ACh transporter (VAChT)-positive cholinergic nerve terminals and adenosine, or its stable analogs, have been reported to reduce nerve-evoked ACh release (26).

An aim of this study was to measure directly in mouse detrusor the effect of A_1_ receptor agonists on nerve-mediated ATP and ACh release, as well as tension generation, to determine any differential effect on neurotransmitter release. A further aim was to characterize downstream cAMP-dependent pathways involved in any such actions. The motivation of the study was to identify potential drug targets that may selectively attenuate the release of transmitters associated with DO in humans.

## MATERIALS AND METHODS

### Tissue Samples and Ethics Approval

All animal care and experimental procedures followed the University of Bristol Ethics Committee guidelines and were in accordance with United Kingdom legislation under the Animals (Scientific Procedures) Act 1986 Amendment Regulations (SI 2012/3039) and the principles of the National Institutes of Health (NIH). Young (12 wk) male C57BL/6 mice (Harlan UK, Ltd.) were used for experiments. The animal model was chosen to conform with previous experiments where transmitter release methods were validated (27, 28) and according to the stipulations of the funding authority (NIH).

### Measurement of Contractile Function In Vitro

Mice were killed by CO_2_ asphyxiation and the bladder was removed through a midline laparotomy. The whole bladder was bisected and bladder strips from the bladder dome (detrusor with mucosa intact, 4–5 mm length, 1–2 mm width) were tied in a horizontal trough between a hook and an isometric force transducer. Preparations were superfused with Tyrode solution at 37°C. Contractions generated by electrical field stimulation (EFS; 0.1 ms pulses, 1–40 Hz, 3-s train every 90 s) were inhibited by tetrodotoxin (TTX, 1 µM). Drugs were added to the superfusate, with appropriate vehicle and time controls, and the effects on nerve-mediated contraction amplitude were measured. Tension (mN) was normalized to preparation weight (mN·mg^−1^) to avoid confounding experimental variability due to preparation dimensions.

### Measurement of Nerve-Mediated Neurotransmitter Release

Superfusate samples (100 µL) were taken from a fixed point near the preparation (two-thirds downstream along the tissue length and 1 mm lateral to the horizontally mounted preparation), ensuring minimal mechanical disturbance. Samples were taken before EFS, and 2 s after the initiation of EFS, with nerve-mediated release taken as the difference between these two values. Samples were stored on ice before assay of released ATP or ACh. In separate experiments, EFS-mediated ATP and ACh release was completely inhibited by TTX (1 µM) or 2% lignocaine (*n* = 6 each).

#### Measurement of sampled ATP.

ATP release was measured using a luciferin-luciferase assay where emitted light was a positive function of ATP concentration. The complete Sigma ATP assay mix (FLAAM, Sigma-Aldrich, Dorset, UK) was diluted with an assay buffer supplied, as per the manufacturer’s instructions. Luminescence intensity was read using a luminometer (Glomax 20/20, Promega) and calibrated with an ATP standard on the day of each experiment, with luminescence being a linear function of concentration on a log-log plot over the range of 100 fM to 1 µM. A log-log plot was chosen to linearize the calibration curve over the wide range of calibration solution concentrations. The detection limit of the system was 100 fM ATP. ATP release was measured across the EFS frequency range to elicit contractions.

#### Measurement of sampled ACh.

ACh release was measured with a choline/ACh quantification assay (MAK056, Sigma-Aldrich, Dorset, UK) using the fluorescence method following the manufacturer’s instructions. Reaction mixes were added to collected samples (50 µL)—one with acetylcholinesterase (AChE) added to the reaction mix, which hydrolyzes ACh to choline and acetate to determine total choline, and the other without AChE to determine free, background choline levels. The difference between the two was equivalent to the quantity of ACh from nerve-mediated stimulation. Fluorescence intensity (λ_ex_ = 535/λ_em_ = 587 nm) was read using a fluorescence multiwell plate reader (CLARIOstar Plus, BMG Labtech). The system was calibrated with a choline standard on the day of each experiment, with fluorescence a linear function of concentration over the range of 0–250 pM choline.

### Data and Statistical Analyses

**Figure 1.**
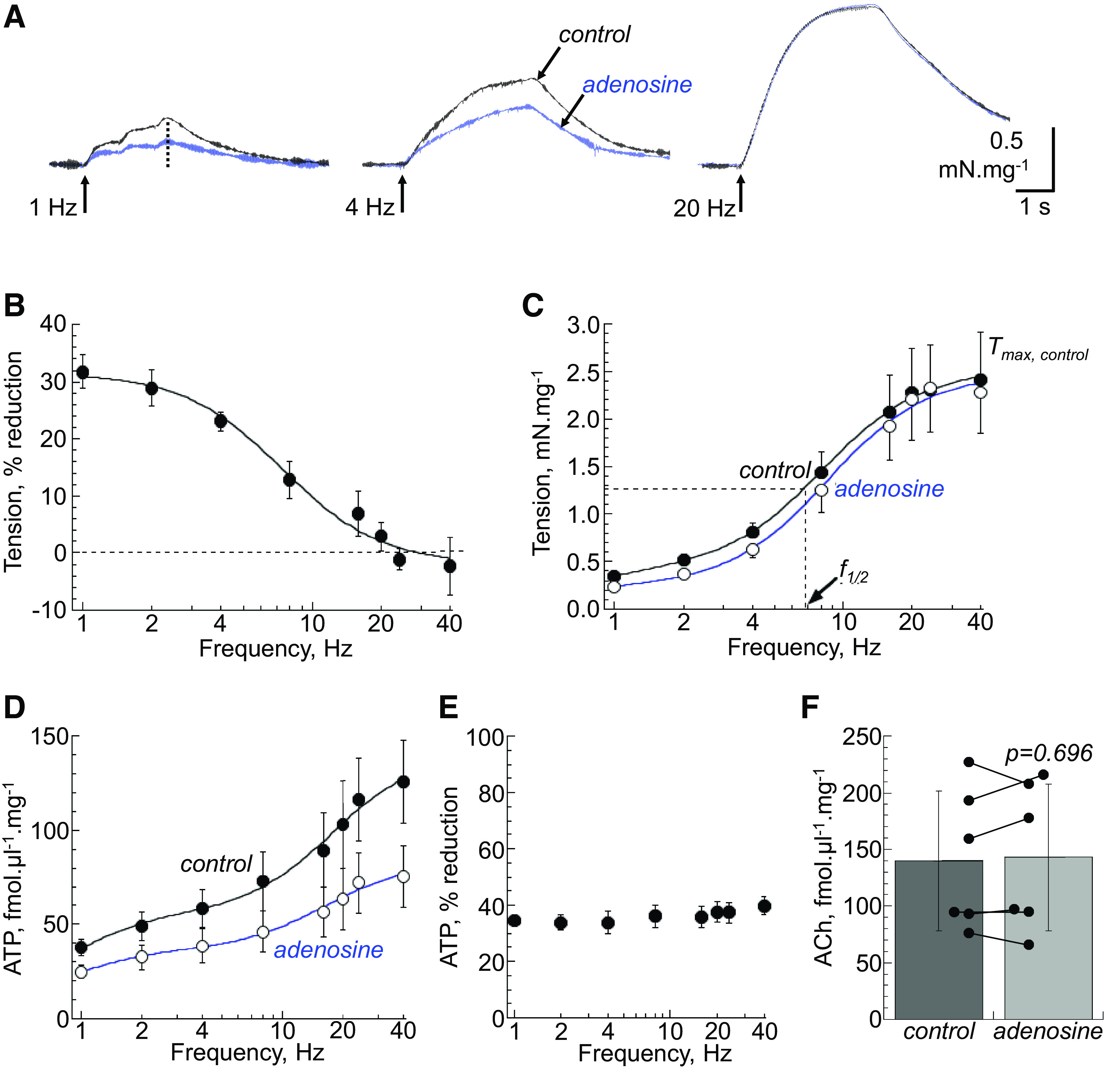
*A*: representative traces of nerve-mediated contractions under control conditions and with adenosine (1 mM, *n* = 12) at 1, 4, and 20 Hz stimulation. At 1 Hz stimulation, the response is measured from the final transient of the individual stimuli (dotted line). *B*: percentage reduction of nerve-mediated contractions by adenosine. Fits are from *Eq. 1a*, materials and methods. *C*: force-frequency relationship curves for nerve-mediated contractions in control and in the presence of adenosine. Fits are from *Eq. 1b*. Parameters measured include *T*_max_ (mN·mg^−1^) and *f*_1/2_ (Hz) and are included in Table 2. *D*: ATP release-frequency relationship curves for nerve-mediated transmitter release in control and in the presence of adenosine. Fits are from *Eq. 1b*. *E*: percentage reduction of nerve-mediated ATP release by adenosine. Fits are from *Eq. 1a*. *F*: adenosine had no effect on nerve-mediated acetylcholine (ACh) release (fmol·µL^−1^·mg^−1^) at 20 Hz stimulation (*n* = 6, Student’s paired *t* test). Individual data points for data sets are illustrated in Supplemental Fig. S1.

The frequency-dependent percentage reduction of tension data, *T*(*f*), by interventions (e.g., Fig. 1*B*) was fitted to *Eq. 1a*

(*1a*) $$T\left(f\right)=T_{\text{Lf}}-\;\left[\left(\frac{\left(T_{\text{Lf}}-T_{\text{Hf}}\right)\cdot f^{m}}{f^{m}+k^{m}}\right)+\;T_{\text{Hf}}\right]$$

*T*_Lf_ and *T*_Hf_ are the maximum and minimum force reductions respectively at low and high frequencies, *m* and *k* are constants.

The frequency dependence of peak tension or ATP release (e.g., Fig. 1, *C* and *D*) were fitted to a linear two-component function, *Eq. 1b*, as this yielded a significantly better fit than a one-component function (28): *Y*(*f*) is equivalent to either *T*(*f*) or [ATP](*f*).

(*1b*) $$Y\left(f\right)=\;\frac{Y_{\text{Lf},max\;}\cdot f^{m}}{f_{\text{*},\text{Lf}}^{m}+f^{m}}+\frac{Y_{\text{Hf},max}\cdot f^{m}}{f_{\text{*},\text{Hf}}^{m}+f^{m}}\;$$

*Y*_max_ are the maximum estimated values of low (Lf) or high (Hf) components, and f_*_ are the frequencies at which the two components each reach *Y*_max/2_; *m* is a constant. The *T*_max_ is the sum of the *T*_max_ values of Lf and Hf components, and *f*_1/2_ is the frequency at half-maximal tension, *T*_max/2_—each of which is reported in Tables 2 and 3.

Frequency-dependent ACh data were fitted to a one-component function, *Eq. 1c*; there was no statistical advantage by fitting ACh data to a two-component model (28).

(*1c*) $$\left[\text{ACh}\right]\left(f\right)=\;\frac{Y{\left[\text{ACh}\right]}_{\text{max}}\cdot f^{m}}{f_{\frac{1}{2}}^{m}+\;f^{m}}$$

*Y*_max_ is the maximum estimated value of *Y* and *f*_1/2_ is the frequency required to achieve *Y*_max/2_; *m* is a constant.

Data fits were performed with an iterative, least-squares Levenberg–Marquardt algorithm (KaleidaGraph, v4.5, Synergy software). Data are means ± SD and differences between data sets were tested with Student’s paired *t* tests, repeated-measures one-way ANOVA followed by parametric post hoc tests, and repeated-measures two-way ANOVA followed by parametric post hoc tests where appropriate; the null hypothesis was rejected at *P* < 0.05. *n* values refer to the number of preparations, one each from separate animals.

Values of *Y*_Lf,max_ (*Eq. 1b*) were used to analyze specifically the effect of interventions on purinergic contractions without the involvement of nerve-mediated ACh release. This component was dominant over the frequency range over 1–2 Hz (f_*_,_Lf_ = 0.7 ± 0.2 Hz, *n* = 48), where ACh release was negligible. An association between two variables, *r*, was calculated as a Pearson correlation coefficient. All statistical analyses were undertaken using GraphPad Prism 7 (GraphPad Software, Inc.; GraphPad Prism). The number of repeats in each control and intervention set was based on a power calculation to reject the null hypothesis at *P* < 0.05 and a power of 80%, with a variance of data based on previous experimental data with these methods. The data and statistical analyses comply with the guidelines for reporting statistics in journals published by the American Physiological Society (29).

### Materials

Tyrode solution was composed of the following (mM): 118 NaCl, 24 NaHCO_3_,4.0 KCl, 0.4 NaH_2_PO_4_, 1.0 MgCl_2_, 1.8 CaCl_2_,6.1 glucose, 5.0 Na pyruvate; 5% CO_2_-95% O_2_, pH 7.4.

The concentration of all stock solutions was between 1.0 and 10 mM. Adenosine, atropine, cyclic 3′,5′-(hydrogenphosphorothioate) triethylammonium (cAMPS-Rp), *N*^6^-monobutyryladenosine 3′,5′-cyclic monophosphate sodium salt (6-MB-cAMP), 8-(4-chlorophenylthio)-2′-*O*-methyladenosine-3′,5′-cyclic monophosphate acetoxymethyl ester (007-AM), and α-[2-(3-chlorophenyl)hydrazinylidene]-5-(1,1-dimethylethyl)-*b*-oxo-3-isoxazolepropanenitrile (ESI-09) were dissolved in distilled water. CPA, 5′-(*N*-ethylcarboxamido)adenosine (NECA), and 8-cyclopentyl-1,3-dipropylxanthine (DPCPX) were dissolved in DMSO. Forskolin was dissolved in ethanol. Stock solutions were diluted with Tyrode solution to the final concentration as indicated. Adenosine, atropine, CPA, NECA, DPCPX, forskolin, 6-MB-cAMP, and ESI-09 were from Sigma-Aldrich (Dorset, UK), and cAMPS-Rp and 007-AM were from Tocris (Abingdon, UK). The mechanism of action of drugs and the concentrations used in this study are listed in Table 1.

**Table 1.** Mechanism of action of drugs used in this study

Drug	Mechanism of Action	Concentration Used
Adenosine	Endogenous adenosine receptor agonist	1 mM
CPA	Selective adenosine A_1_ receptor agonist	10 µM
NECA	Adenosine A_1_/A_2_ receptor agonist	10 µM
DPCPX	Selective adenosine A_1_ receptor antagonist	1 µM
Forskolin	AC activator	1 µM
Atropine	Non-selective muscarinic receptor antagonist	1 µM
cAMPs-Rp	Selective PKA inhibitor (cell permeable)	10 µM
6-MB-cAMP	PKA activator	100 µM
007-AM	EPAC activator	10 µM
ESI-09	EPAC inhibitor	20 µM

007-AM, 8-(4-chlorophenylthio)-2′-*O*-methyladenosine-3′,5′-cyclic monophosphate acetoxymethyl ester; cAMPS-Rp, 3′,5′-(hydrogenphosphorothioate) triethylammonium; CPA, *N*^6^-cyclopentyladenosine; DPCPX, 8-cyclopentyl-1,3-dipropylxanthine; EPACs, exchange protein activated by cAMP; ESI-09, α-[2-(3-chlorophenyl)hydrazinylidene]-5-(1,1-dimethylethyl)-*b*-oxo-3-isoxazolepropanenitrile; 6-MB-cAMP, *N*^6^-monobutyryladenosine 3′,5′-cyclic monophosphate sodium salt; NECA, 5′-(*N*-ethylcarboxamido)adenosine.

## RESULTS

### Adenosine on Nerve-Mediated Contractions and Neurotransmitter Release

Adenosine (1 mM) reduced nerve-mediated contractions, but the effect was frequency dependent, with a reduction by ∼30% at 1 Hz, but absent at 20 Hz (Fig. 1, *A* and *B*). The frequency-dependent effect was quantified in two ways. First, by plotting the percentage reduction of force as a function of frequency (Fig. 1*B*) and fitting the data with *Eq. 1a*, materials and methods, to show a maximal reduction of 31.8 ± 7.2% at low frequencies and a half-maximal reduction at 6.7 ± 2.6 Hz. The second approach was to generate separate force-frequency relations using *Eq. 1b* (Fig. 1*C*) to obtain parameters that are shown in Table 2, the estimated *T*_max_ at high frequencies; and the frequency to achieve *T*_max_/2, *f*_1/2_. Adenosine had no effect on *T*_max_ but increased *f*_1/2_, consistent with a preferential reduction of force at low frequencies. The first approach is illustrated in subsequent figures, where relevant. However, because quantitative data were not possible when there was no effect of the intervention, the second approach provided parameterized data to demonstrate an effect, or otherwise, of an intervention (Table 2).

**Table 2.** Force-frequency curve parameters T_max_ and f_1/2_ with modulation of cAMP-dependent signaling pathways

Intervention	*T*_max_, mN·mg^−1^	*P*	*f*_1/2_, Hz	*P*
Control (*n* = 12)	2.18 ± 0.66		5.1 ± 1.3	
+ adenosine	2.19 ± 0.61	0.893	7.5 ± 2.0**	0.002
Control (*n* = 6)	1.81 ± 0.41		4.0 ± 2.2	
+ DPCPX	1.96 ± 0.35	0.068	4.4 ± 1.4	0.218
+ DPCPX, aden.	2.01 ± 0.34	0.410	4.7 ± 0.8	0.423
Control (*n* = 6)	1.92 ± 0.60		5.8 ± 1.5	
+ CPA	1.95 ± 0.67	0.077	9.0 ± 2.4***	0.0002
Control (*n* = 6)	1.94 ± 0.56		5.8 ± 1.5	
+ NECA	2.12 ± 0.57	0.124	10.7 ± 2.3**	0.001
Control (*n* = 6)	1.99 ± 0.55		5.9 ± 1.4	
+ cAMPs-Rp	2.15 ± 0.54**	0.006	7.4 ± 1.3**	0.006
Control (*n* = 8)	2.09 ± 0.30		6.6 ± 1.6	
+ ESI-09	2.22 ± 0.42	0.293	6.7 ± 1.1	0.826
Control (*n* = 6)	2.24 ± 0.31		5.6 ± 0.8	
+ 6-MB-cAM*P*	2.26 ± 0.39	0.814	5.9 ± 1.4	0.730
+ 6-MB-cAMP, aden.	2.37 ± 0.36	0.150	8.5 ± 1.8*	0.026
Control (*n* = 6)	2.60 ± 0.72		6.6 ± 1.3	
+ 007-AM	2.86 ± 1.06	0.062	5.9 ± 1.4	0.479
+ 007-AM, aden.	2.73 ± 0.82	0.369	9.1 ± 1.7*	0.0001
Control (*n* = 6)	2.16 ± 0.17		5.5 ± 0.9	
+ forskolin (FSK)	2.30 ± 0.21	0.129	10.5 ± 3.1*	0.019
+ FSK, aden.	2.20 ± 0.34	0.731	19.4 ± 7.7**	0.008
Control (*n* = 6)	1.84 ± 0.46		3.9 ± 0.4	
+ atropine	1.21 ± 0.32**	0.003	2.4 ± 0.1**	0.002
+ atropine, FSK	0.60 ± 0.17**	0.002	2.2 ± 0.6**	0.010

Data are means ± SD. Individual data points in Supplemental Figs. S1–S6. 007-AM, 8-(4-chlorophenylthio)-2′-*O*-methyladenosine-3′,5′-cyclic monophosphate acetoxymethyl ester; cAMPS-Rp, 3′,5′-(hydrogenphosphorothioate) triethylammonium; 007-AM, 8-(4-chlorophenylthio)-2′-*O*-methyladenosine-3′,5′-cyclic monophosphate acetoxymethyl ester; CPA, *N*^6^-cyclopentyladenosine; DPCPX, 8-cyclopentyl-1,3-dipropylxanthine; ESI-09, α-[2-(3-chlorophenyl)hydrazinylidene]-5-(1,1-dimethylethyl)-*b*-oxo-3-isoxazolepropanenitrile; 6-MB-cAMP, *N*^6^-monobutyryladenosine 3′,5′-cyclic monophosphate sodium salt; NECA, 5′-(*N*-ethylcarboxamido)adenosine.

* *P* < 0.05; **P < 0.01; ***P < 0.001 with respect to control or immediately preceding intervention, exact *P* values are also shown (repeated-measures ANOVA followed by parametric post hoc test, see materials and methods).

Neurotransmitter release was measured directly by measuring [ATP] or [ACh] near the preparation, at the same time as contractions were recorded. Control experiments showed that ATP and ACh release, as well as tension, were completely abolished by 1 µM tetrodotoxin at all frequencies used (*n* = 6) and were therefore designated nerve-mediated phenomena. ATP release occurred over the entire frequency range (1–40 Hz) and adenosine reduced ATP release at all frequencies; e.g., at 8 Hz from 73.6 ± 16.3 to 44.2 ± 9.3 fmol·µL^−1^·mg^−1^, a 35.2 ± 6.5% reduction (*n* = 12; Fig. 1*D*). The proportional reduction was similar at all stimulation frequencies (Fig. 1*E*).

ACh release occurred over a different frequency range (>4 Hz to a maximum at 20–40 Hz). Adenosine had no effect on ACh release; release values at 20-Hz stimulation were 143 ± 65 fmol·µL^−1^·mg^−1^ compared with 140 ± 62 fmol·µL^−1^·mg^−1^ at control; *n* = 6, *P* = 0.696 (Fig. 1*F*). Thus, ATP and ACh release occur over different frequency domains; at low frequencies (<4 Hz) ATP release is dominant, with increasing ACh release at higher frequencies. Because adenosine predominantly reduced nerve-mediated tension at lower frequencies, it may be hypothesized that this results from a differential effect on nerve-mediated ATP over ACh release. Henceforth, with adenosine and subsequent interventions, changes to nerve-mediated tension and ATP release will use averaged data at 1 and 2 Hz stimulation, and data for any actions on ACh release will be reported at 20 Hz. Thus, adenosine reduced tension by 29.8 ± 6.6% and ATP release by 34.1 ± 6.8%, with no significant effect on ACh release (Table 3).

**Table 3.** Percentage reduction (red^n^) of nerve-mediated contractions and ATP/ACh release, with modulation of cAMP-dependent signaling pathways

Intervention	*n*	Tension, %Red^n^ 1, 2 Hz	*P*	ATP, %Red^n^ 1, 2 Hz	*P*	ACh, %Red^n^ 20 Hz	*P*
Adenosine	12; 6 ACh	29.8 ± 6.6***	<0.0001	34.1 ± 6.8***	<0.0001	−1.0 ± 10.5	0.697
DPCPX	6	−2.2 ± 6.7	0.539	−0.3 ± 1.7	0.469	Not recorded	
DPCPX, aden.		2.1 ± 12.1	0.524	2.2 ± 4.5	0.420		
CPA	6	50.7 ± 5.7**	0.003	44.1 ± 4.4***	<0.0001	−5.6 ± 7.2	0.119
NECA	6	69.2 ± 9.1***	0.0005	51.8 ± 8.6 ***	<0.0001	−0.7 ± 10.2	0.983
cAMPs-Rp	6	46.4 ± 2.4**	0.002	35.5 ± 3.2***	<0.0001	0.4 ± 5.1	0.469
ESI-09	6	0.1 ± 11.5	0.900	−1.2 ± 2.4	0.334	0.4 ± 9.9	0.962
6-MB-cAMP	6	3.1 ± 2.6	0.173	−3.0 ± 3.3	0.349	Not recorded	
6-MB-cAMP, aden.		41.4 ± 18.4**	0.008	32.1 ± 4.4***	0.0002		
007-AM	6	4.3 ± 1.2	0.251	−0.7 ± 3.2	0.482	Not recorded	
007-AM, aden.		34.0 ± 2.8**	0.004	30.3 ± 8.3*	0.014		

Data are means ± SD. Reductions are referenced to control conditions, and in cases when adenosine is also added after an intervention, with reference to that preceding intervention. Tension and ATP values are the averaged reductions at 1 and 2 Hz stimulation. Acetylcholine (ACh) values are as recorded at 20 Hz stimulation. 007-AM, 8-(4-chlorophenylthio)-2′-*O*-methyladenosine-3′,5′-cyclic monophosphate acetoxymethyl ester; CPA, *N*^6^-cyclopentyladenosine; DPCPX, 8-cyclopentyl-1,3-dipropylxanthine; ESI-09, α-[2-(3-chlorophenyl)hydrazinylidene]-5-(1,1-dimethylethyl)-*b*-oxo-3-isoxazolepropanenitrile; 6-MB-cAMP, *N*^6^-monobutyryladenosine 3′,5′-cyclic monophosphate sodium salt; NECA, 5′-(*N*-ethylcarboxamido)adenosine

* *P* < 0.05, **P < 0.01; ***P < 0.001 with respect to control or immediately preceding intervention, exact *P* values are also shown. Individual data points in Supplemental Figs. S1–S6.

### A_1_ Receptor Modulation, Nerve-Mediated Contractions, and ATP/ACh Release

The selective A_1_ receptor antagonist DPCPX (1 µM) alone had no effect on tension or on ATP release at any frequency (Tables 2 and 3). Furthermore, in the presence of DPCPX, there was no effect of adenosine on nerve-mediated contractions and ATP release (Tables 2 and 3). This is consistent with the involvement of the A_1_ receptor whereby adenosine suppresses nerve-mediated tension and ATP release.

**Figure 2.**
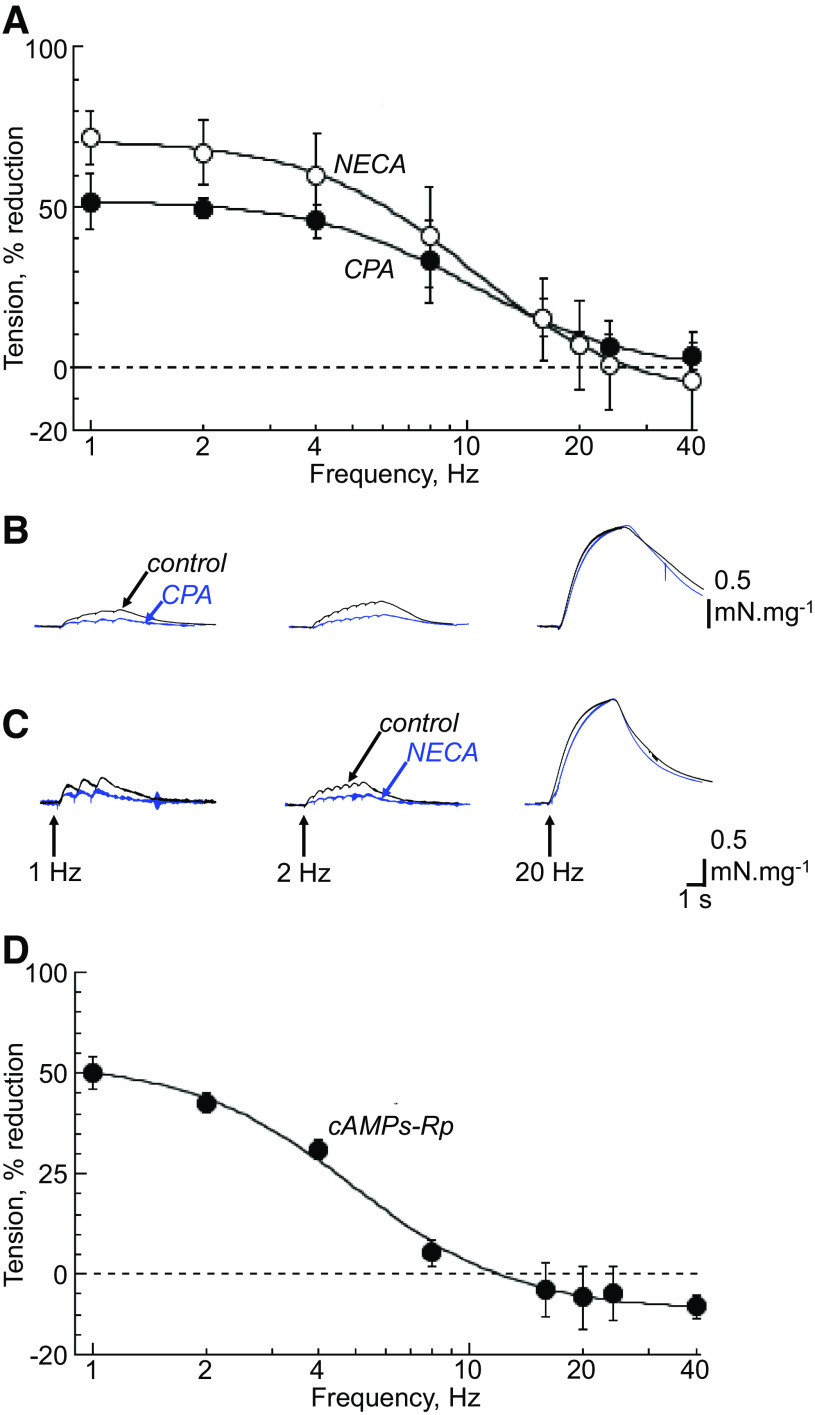
*A*: percentage reduction of nerve-mediated contractions by *N*^6^-cyclopentyladenosine (CPA) (10 µM, *n* = 6) and 5'-(*N*-ethylcarboxamido)adenosine (NECA) (10 µM, *n* = 6). Fits are from *Eq. 1a*. Representative traces of nerve-mediated contractions under control conditions and with CPA (*B*) and NECA (*C*), at 1, 2 and 20 Hz stimulation. *D*: percentage reduction of nerve-mediated contractions by 3',5'-(hydrogenphosphorothioate) triethylammonium (cAMPs-Rp) (10 µM, *n* = 6). Fits are from *Eq. 1a*.

The selective A_1_ receptor agonist, CPA (10 µM), and the less-selective A_1_/A_2_ receptor agonist, NECA (10 µM) also reduced nerve-mediated contractions, predominantly at low frequencies, with a maximal reduction of 54.3 ± 6.13% and 73.8 ± 10.5%, respectively (Fig. 2, *A*–*C*). The half-maximal reduction was at 10.0 ± 3.5 Hz for CPA, and 10.4 ± 3.6 Hz for NECA (Fig. 2*A*). With both receptor agonists there was no effect on the *T*_max_, and an increase of the *f*_1/2_ values (Table 2), as well as reduction of low-frequency nerve-mediated contractions and ATP release, but no effects on ACh release (Table 3).

### Downregulation of cAMP-Dependent Signaling Pathways, Nerve-Mediated Contractions, and ATP/ACh Release

Activation of adenosine A_1_ receptors reduces AC activity to reduce intracellular cAMP levels, and it was further hypothesized that this mediates the aforementioned A_1_-receptor actions on nerve-mediated tension and ATP/ACh release. PKA and EPAC are targets for cAMP and the roles of each were investigated with selective modulators. The PKA antagonist, cAMPS-Rp (10 µM), had overall effects similar to adenosine or CPA. There was a selective reduction of low-frequency contractions (Fig. 2*D*), as well as an increase of *f*_1/2_ (Table 2), and in this case, a small but significant increase of *T*_max_. In addition, low-frequency contractions and nerve-mediated ATP release were attenuated with no effect on ACh release (Table 3).

A role for EPAC was explored using the inhibitor, ESI-09 (20 µM). There was no effect on magnitude or frequency dependence of nerve-mediated contractions nor on corresponding ATP or ACh release (Tables 2 and 3). Thus, the principal target for altered intracellular cAMP levels to regulate tension or transmitter release is on this evidence via PKA.

**Figure 3.**
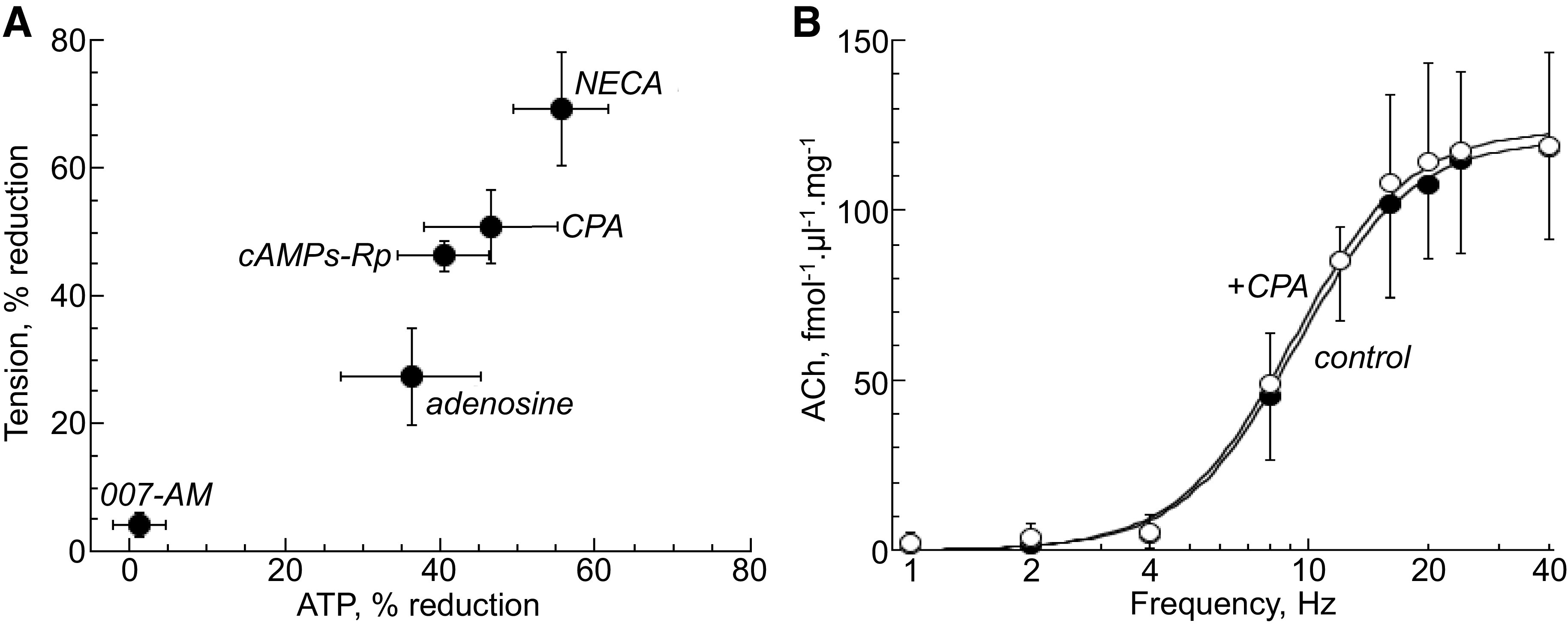
*A*: relationship between tension and ATP release at low stimulation frequencies. Values of *Y*_Lf,max_ from *Eq. 1b*. were used to analyze the effect of interventions on purinergic contractions without the involvement of nerve-mediated acetylcholine (ACh) release, specifically at 1 and 2 Hz. There is a significant (*r* = 0.97, *P* = 0.001) relationship between reduction of both tension and ATP release at low stimulation frequencies. *B*: at low frequencies, no nerve-mediated ACh release was recorded. In this example, *N*^6^-cyclopentyladenosine (CPA) (10 µM, *n* = 6) had no effect on nerve-mediated ACh release across the frequency range. Fits are from *Eq. 1c*. cAMPs-Rp, 3',5'-(hydrogenphosphorothioate) triethylammonium; NECA, 5'-(*N*-ethylcarboxamido)adenosine.

Figure 3*A* summarizes the aforementioned experiments with adenosine, CPA, NECA, and cAMPs-Rp to demonstrate a significant (*r* = 0.97, *P* = 0.001) relationship between the reduction of both tension and ATP release at low stimulation frequencies (Table 3). Included, also are data from the EPAC activator, 007-AM that exerted no effect on either variable. There was also a significant order of potency for the A_1_-receptor agonists, with NECA > CPA adenosine. These data are consistent with an adenosine A_1_ receptor-mediated pathway via PKA to suppress selectively both nerve-mediated ATP release and low-frequency contractions.

The selective adenosine A_1_ receptor agonist, CPA, reduced atropine (1 µM)-resistant, purinergic contractions by 26%–44% across the frequency range (1–40 Hz, *n* = 6). Similarly, CPA resulted in a 30%–45% reduction of ATP release across the same frequencies (*n* = 6). It is worth noting that this proportional reduction of atropine-resistant contractions and nerve-mediated ATP release was consistent at all frequencies—i.e., they were not frequency dependent, similar to the inhibitory effect of adenosine on nerve-mediated ATP release (Fig. 1*E*).

### Upregulation of cAMP-Dependent Signaling Pathways, Nerve-Mediated Contractions, and ATP/ACh Release

By contrast, interventions designed to upregulate cAMP signaling have no significant effect on nerve-mediated tension or ATP/ACh release. The cAMP analog, 6-MB-cAMP (100 µM), a PKA activator, had no effects on nerve-mediated contractions or nerve-mediated ATP release (Tables 2 and 3). In addition, the EPAC activator, 007-AM (10 µM), also had no effects on contraction magnitude or ATP/ACh release at any frequency (Tables 2 and 3). However, in both cases, the subsequent addition of adenosine in the continuous presence of 6-MB-cAMP or 007-AM produced comparable reductions of contraction magnitude and ATP release as generated by adenosine alone (Tables 2 and 3).

**Figure 4.**
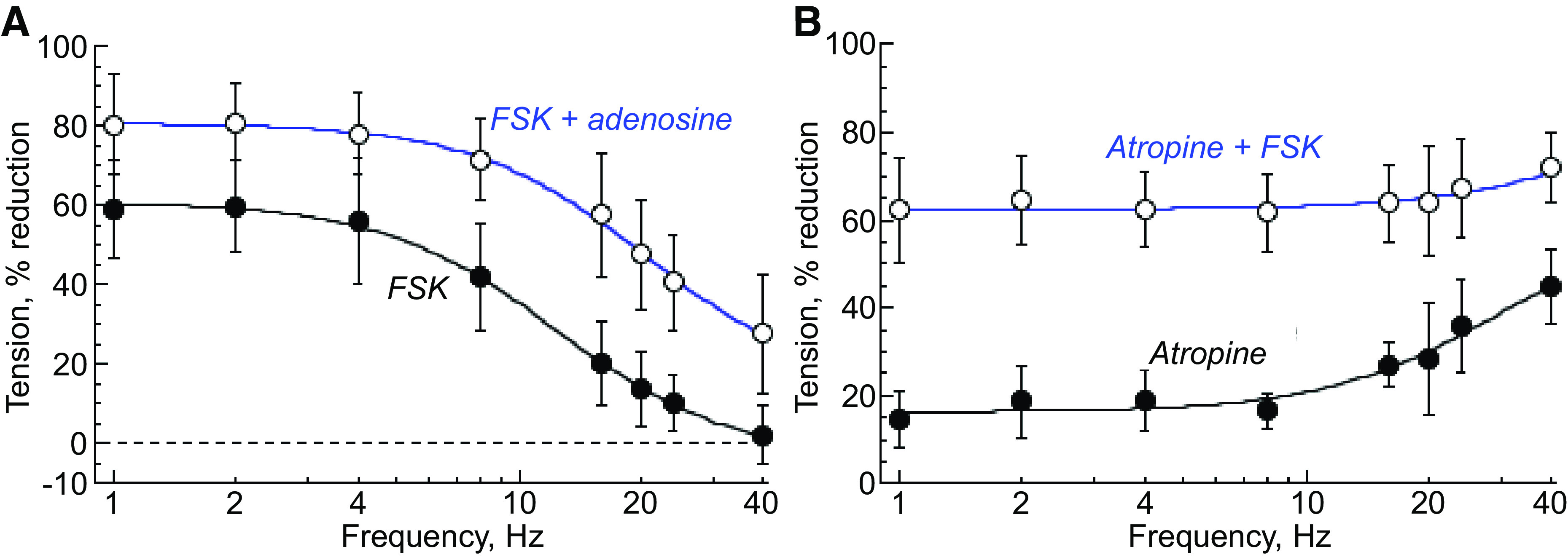
*A*: percentage reduction of nerve-mediated contractions by forskolin (1 µM) and adenosine (1 mM) in the presence of forskolin (*n* = 6). *B*: percentage reduction of nerve-mediated contractions by atropine (1 µM) and forskolin (1 µM) in the presence of atropine (*n* = 6). Fits are from *Eq. 1b.* FSK, forskolin.

The AC activator, forskolin (1 µM), reduced nerve-mediated contractions, with a maximal reduction of 61.6 ± 12.4% at low frequencies and a half-maximal reduction at 13.2 ± 4.6 Hz (Fig. 4*A*). However, it had no effect on nerve-mediated ATP or ACh release (Supplemental Fig. S6; all Supplemental Figures are available at https://doi.org/10.6084/m9.figshare.21253557). Adenosine in the presence of forskolin produced an additional effect, further reducing low-frequency contractions (Fig. 4*A*) and nerve-mediated ATP release (Table 3). Forskolin was the only intervention that also reduced the resting baseline tension, by 25.5 ± 17.4% (Student’s paired *t* test). This effect was significant compared with other interventions, for example, adenosine, had no effect on resting baseline tension (8.9 ± 14.4%, two-way ANOVA, Supplemental Fig. S6). Finally, forskolin directly reduced purinergic contractions (Fig. 4*B*). Atropine (1 µM) reduced nerve-mediated contractions, with a reduction of *T*_max_ and *f*_1/2_ values, and these atropine-resistant contractions were further reduced by forskolin (Table 2).

## DISCUSSION

### Adenosine Receptor Pathways in Nerve-Mediated Tension and Neurotransmitter Release

Adenosine reduces nerve-mediated contractions in the detrusor of many species including those from mice (20) and humans (6). In humans, adenosine had a greater reduction in detrusor nerve-mediated contractions from patients with DO compared with those from normal bladders. In addition, there was a greater effect at lower stimulation frequencies in these pathological bladders (6). Nerve-mediated contractions are generated by ATP and ACh release from efferent nerves, with dominant roles for the purinergic component of release at lower frequencies of stimulation and the cholinergic component at higher frequencies (27, 30). The selective A_1_ receptor agonist, CPA, had a similar effect to that of adenosine, and it has been inferred that adenosine acting at adenosine A_1_ receptors suppresses ATP release (21), an action directly confirmed here for the first time. These effects of adenosine were inhibited by the A_1_ receptor antagonist, DPCPX. Adenosine and CPA also reduce the amplitude of excitatory junction potentials (EJPs) in mouse detrusor bladder preparations, mediated by A_1_ receptors and suggest a presynaptic inhibitory effect of A_1_ receptor activation on evoked ATP release (31).

The A_1_/A_2_ receptor agonist, NECA, had a similar effect to CPA, with no suppression of contractions by high-frequency stimulation, a uniform proportional suppression of nerve-mediated ATP release over the range of stimulation frequencies, and no effect on ACh release. There is a differential distribution of A_1_ and A_2_ receptors in the detrusor, with A_2_ receptors more abundant on the detrusor muscle (20, 32, 33). The similarity of actions with CPA and NECA is consistent with their major actions being on prejunctional A_1_ receptors, and consistent, under the conditions of these experiments, with only minor direct actions of adenosine on detrusor via A_2A_ and A_3_ receptors (6).

However, a more complex role for adenosine and A_1_ receptors has been suggested in addition to the reduction of nerve-mediated ATP release, namely to regulate nerve-mediated ACh release as judged from colocalization of A_1_ receptors with vesicular ACh transporters on cholinergic nerve terminals (26). In detailed studies, it was proposed that β_3_-adrenoceptor agonists indirectly mediate adenosine release from detrusor smooth muscle, via the equilibrative nucleoside transporter 1, to activate A_1_ receptors on cholinergic nerves and reduce neuronal ACh release (34, 35). These varying effects of adenosine on nerve-mediated ACh release may be due to the difference in the concentration of adenosine and the different conditions from the experiments reported here. The aforementioned observation is important as a potential additional route whereby β_3_-adrenoceptor agonists may relax detrusor smooth muscle.

Adenosine may also exert actions at other sites in the urinary bladder and contribute to its overall effects. Spontaneous contractions in isolated detrusor smooth muscle are accompanied by similar ATP release transients from motor nerves (36) and may represent leakage from the two vesicular pools measured in this study. However, A_1_ receptor involvement was not supported by the fact that neither adenosine nor CPA altered spontaneous contractions or accompanying EJPs (31). In addition, the mucosa is another source of ATP, evoked by mechanical or chemical stimuli (37, 38), in turn, increased in tissue from patients with OAB (39). The neurotoxin, TTX, completely abolished EFS-induced ATP release at all frequencies in these experiments. With rat mucosal strips, EFS-induced ATP release was unaffected by TTX, except for small effects at 20 and 40 Hz, and suggests a minor role for mucosal ATP release in these experiments (38).

All adenosine receptors are expressed in the urothelium; however, an adenosine A_1_ receptor agonist is the most potent stimulator of umbrella cell exocytosis, whilst the A_1_ receptor antagonist, DPCPX, was most effective at inhibiting adenosine-induced changes in capacitance. This suggests that the A_1_ receptor is the predominant adenosine receptor regulating transmitter exocytosis at the mucosal surface (40). However, adenosine reduces distension-induced ATP release, and A_1_ receptor antagonism enhanced urothelial ATP release (41). In cystometry studies, the adenosine A_1_ receptor agonist reduced threshold pressure, and intercontraction intervals, which is reversed by DPCPX (42). Intravesical administration of adenosine A_1_ receptor agonist also has an inhibitory effect on the micturition reflex but administration of adenosine A_2_ receptor agonists had no effect (43), suggesting a potential role for adenosine A_1_ receptors in stretch-activated urothelial ATP release and targeting pathological purinergic sensory pathways.

### Cellular Pathways Mediating A_1_ Receptor Activation

**Figure 5.**
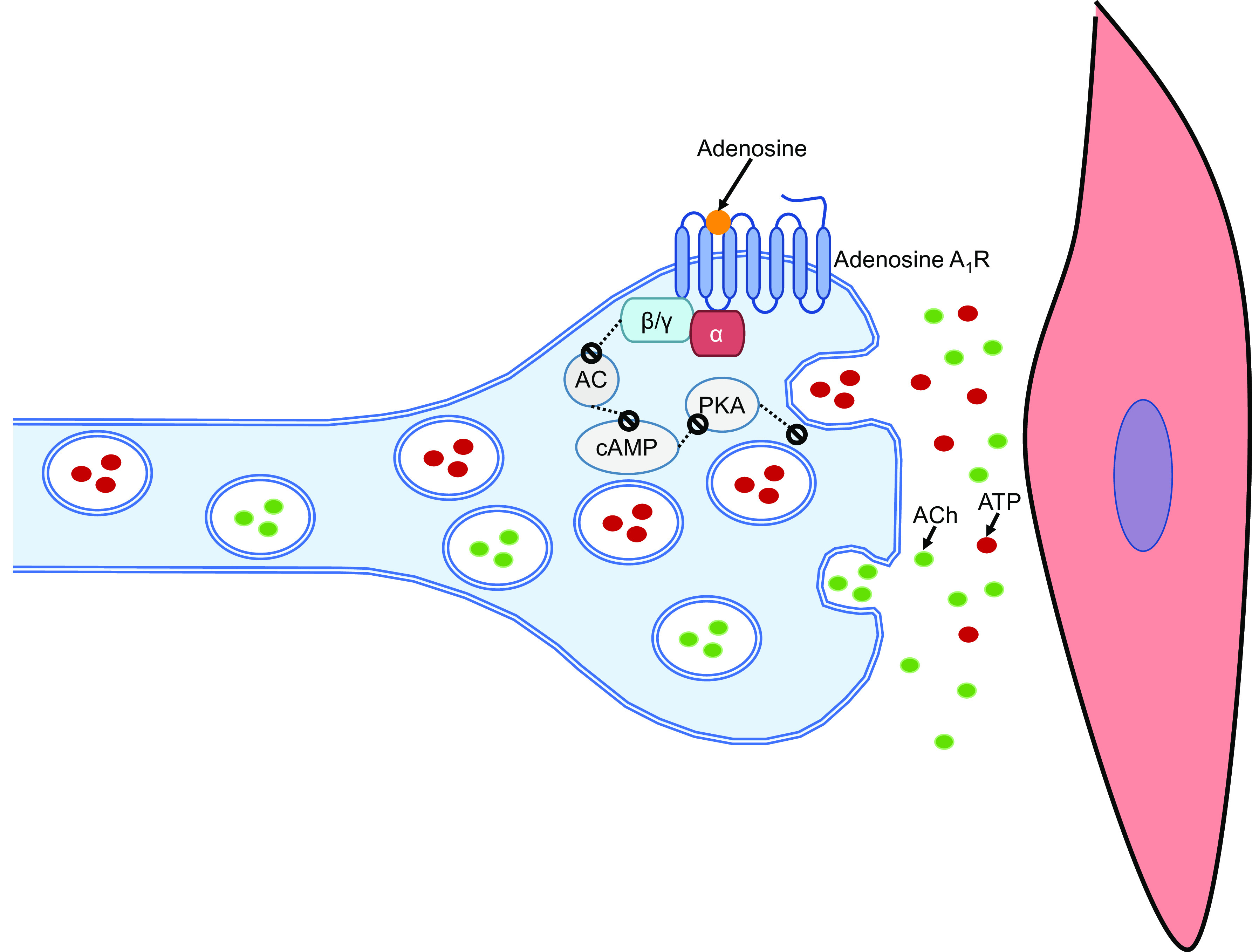
Schematic diagram displaying the prototypical transduction pathway upon the activation of adenosine A_1_ receptors by adenosine. Activation of this receptor inhibits adenylyl cyclase activity and decreases cAMP levels. Downstream signaling pathways involving cAMP signaling include PKA, which plays a role in reducing neuronal ATP release (red) from efferent nerve terminals and the purinergic component of nerve-mediated contractions. Activation of adenosine A_1_ receptors had no effect on neuronal acetylcholine (ACh) release (green) and the cholinergic component of nerve-mediated contractions in the mouse detrusor.

Adenosine A_1_ receptors use the prototypical transduction pathway for the G_i_/G_o_ protein family, inhibiting AC activity and decreasing cAMP levels (19), to reduce the purinergic component of nerve-mediated contractions and neuronal ATP release (Fig. 5). Downstream intracellular pathways involving cAMP signaling include intermediates like PKA and EPACs (19). In this study, the cAMP analog/PKA activator, 6-MB-cAMP, had no effect on neuronal ATP release, and on any parameters of nerve-mediated contractions. The actions of adenosine on nerve-mediated contractions and neuronal ATP release were also mimicked by the PKA inhibitor, cAMPs-Rp. The addition of the EPAC activator, 007-AM, or EPAC inhibitor, ESI-09, had no effect on neuronal ATP release or nerve-mediated contractions. Although inhibiting EPACs reduces the inhibitory effect of forskolin on ACh release (35), inhibiting PKA or EPACs had no direct effect on neuronal ACh release measured in this study. Modulators of cAMP effectors PKA and EPACs have been shown to regulate P_2_X receptors and consequently atropine-resistant, purinergic-mediated contractions of the detrusor (44, 45). This study suggests a role for downstream PKA signaling, and not signaling via EPACs, in the regulation of neuronal ATP release, with subsequent effects on detrusor (Fig. 5). The presence of activators of PKA or EPAC signaling did not have an impact on the ability of adenosine to reduce ATP release from efferent nerve terminals and the low-frequency, purinergic component of nerve-mediated contractions of detrusor smooth muscle in the mouse bladder.

The pathways whereby A_1_ receptor activation reduces neuronal ATP exocytosis, but not ACh, is yet to be determined. Nerve-ending varicosities contain many vesicles enclosing transmitters, that are released via Ca^2+^-dependent exocytosis and Ca^2+^ influx may be mediated by several channel types Ca^2+^ including N-type and P/Q-type channels. With detrusor muscle, it has been proposed that Ca^2+^ entry through N-type channels is associated with ACh release, while P/Q-type channels regulate ATP release (46–49). It is unclear if differential neurotransmitter release is from different populations of nerves, or from different vesicles in the same varicosities. This study suggests that ATP and ACh can be separately released from motor efferent nerves that innervate bladder detrusor smooth muscle, and this is consistent with their different frequency dependencies (28) and the ability to manipulate differential release by modulation of cyclic nucleotides by PDE_5_ inhibitors like sildenafil (27, 28), or adenosine A_1_ receptor activation. It has been demonstrated in several studies that adenosine A_1_ receptor activation can modulate neurotransmitter release (50), suggesting a potential role for the modulation of cyclic nucleotides and downstream effects on protein kinases in the selective inhibition of purinergic neurotransmissions. However, further studies are required to clarify the particular pathways that regulate differential neurotransmitter release from efferent nerves.

Direct application of the AC activator, forskolin, had no effect on the amount of neuronal ATP, nor ACh release, and is consistent with the lack of actions of the cAMP analog, 6-MB-cAMP on ATP release. However, forskolin inhibits the atropine-resistant, purinergic component of nerve-mediated contractions in mouse detrusor strips (44), also confirmed by this study. Forskolin also reduced resting baseline tension, suggesting a direct effect on detrusor smooth muscle and intracellular pathways involved in regulating tension. Detrusor contractions in the mouse bladder induced by the P_2_X receptor agonist, α,β-methylene ATP (α,β-me-ATP), were inhibited by forskolin (44), suggesting a role for AC activation downstream of ATP acting at P_2_X receptors in detrusor smooth muscle. This was further supported by the lack of effect on atropine-resistant nerve-mediated contractions, and α,β-me-ATP-evoked contractions in the mouse detrusor by an AC inhibitor, SQ22536 (44). Downstream intracellular pathways involving cAMP signaling regulate the activity of P_2_X receptors (45). AC is also the target for nonselective activators of G protein-coupled stimulatory pathways, via action on the α subunit. For example, β_3_-adrenoceptor agonists utilize the prototypical pathway for the G_s_ protein family, modulating AC activity and increasing the level of cAMP (19), and several studies have shown an effect of β_3_-adrenoceptor activation on inhibiting both purinergic and cholinergic contractions of the detrusor (44, 51). In this study, activating AC with forskolin did not hinder the ability of adenosine to reduce neuronal ATP release from efferent nerve terminals and further reduce the lower frequency, purinergic component of nerve-mediated contractions of detrusor smooth muscle.

In conclusion, adenosine A_1_ receptor agonism generated a frequency-dependent attenuation of nerve-mediated contractions, with a greater effect at lower stimulation frequencies where ATP release is more predominant, compared with ACh. This was corroborated by direct measurement of reduced nerve-mediated ATP release. By contrast, adenosine had no effect on ACh release or contractions at higher stimulation frequencies. A selective adenosine A_1_ receptor antagonist, DPCPX, abolished the effects of adenosine, consistent with the hypothesis that adenosine acts via A_1_ receptor activation to regulate ATP release from efferent nerve terminals. The main target for cAMP in mediating nerve-mediated ATP release is via PKA and not by an EPAC route. Increasing cAMP levels had no effect, which implies normal levels of intracellular cAMP are sufficient to maintain nerve-mediated ATP release. This study demonstrates that differential regulation of transmitter release is possible at the detrusor nerve-muscle junction. The ability to specifically attenuate ATP release offers a novel therapeutic target, as ATP is associated with pathological contractile function in the human bladder, while leaving physiological contractile function unaffected. Adenosine selectively reduces ATP release from motor nerves supplying detrusor smooth muscle, and the pathway of this action has been characterized: initially by activation of an adenosine A_1_ receptor, with downstream inhibition of AC, cAMP generation, and PKA. Modulation of cyclic nucleotide levels, such as cAMP, provides a novel target of pathological purinergic motor pathways implicated in conditions like OAB.

### Perspectives and Significance

The current first-line of treatment for OAB (antimuscarinics) have recognized limitations, including uncertain efficacy as normal physiological pathways involved in healthy bladder function are suppressed, and adverse side effects. Our study demonstrated a novel finding in the bladder, where adenosine acting via adenosine A_1_ receptor activation, and downstream signaling of cAMP and PKA, selectively inhibits ATP release from motor efferent nerves to detrusor smooth muscle, while leaving ACh release intact. This is of clinical interest as pathological bladders in humans are associated with these pathways—enhanced purinergic motor pathways. Our findings offer the ability to differentially regulate neuronal transmitter release at the neuromuscular junction, with the possibility to suppress pathological pathways, rather than suppressing normal physiological pathways associated with ACh release, as occurs at present with current therapeutic interventions.

## SUPPLEMENTAL DATA

10.6084/m9.figshare.21253557 Supplemental Figs. S1–S6: https://doi.org/10.6084/m9.figshare.21253557.

## GRANTS

Funding was provided by the National Institutes of Health Grant NIH R01 DK098361 (to A. J. Kanai, M. J. Drake, and C. H. Fry).

## DISCLOSURES

No conflicts of interest, financial or otherwise, are declared by the authors.

## AUTHOR CONTRIBUTIONS

B.C. and C.H.F. conceived and designed research; B.C. performed experiments; B.C. and C.H.F. analyzed data; B.C. and C.H.F. interpreted results of experiments; B.C. and C.H.F. prepared figures; B.C. and C.H.F. drafted manuscript; B.C., M.J.D., A.J.K., and C.H.F. edited and revised manuscript; B.C., M.J.D., A.J.K., and C.H.F. approved final version of manuscript.
